# Radiomics Features of the Spleen as Surrogates for CT-Based Lymphoma Diagnosis and Subtype Differentiation

**DOI:** 10.3390/cancers14030713

**Published:** 2022-01-29

**Authors:** Johanna S. Enke, Jan H. Moltz, Melvin D'Anastasi, Wolfgang G. Kunz, Christian Schmidt, Stefan Maurus, Alexander Mühlberg, Alexander Katzmann, Michael Sühling, Horst Hahn, Dominik Nörenberg, Thomas Huber

**Affiliations:** 1 Department of Radiology, University Hospital, LMU Munich, 81377 Munich, Germany; melvin.a.danastasi@gov.mt (M.D.); Wolfgang.Kunz@med.uni-muenchen.de (W.G.K.); Stefan.Maurus@med.uni-muenchen.de (S.M.); Dominik.Noerenberg@medma.uni-heidelberg.de (D.N.); thomas.huber@umm.de (T.H.); 2 Fraunhofer Institute for Digital Medicine MEVIS, 28359 Bremen, Germany; jan.moltz@mevis.fraunhofer.de (J.H.M.); horst.hahn@mevis.fraunhofer.de (H.H.); 3 Medical Imaging Department, Mater Dei Hospital, 2090 MSD Msida, Malta; 4 Department of Medicine III, University Hospital, LMU Munich, 81377 Munich, Germany; Christian_Schmidt@med.uni-muenchen.de; 5 CT R&D Image Analytics, Siemens Healthineers, 91301 Forchheim, Germany; alexander-muehlberg@hotmail.com (A.M.); alexander.katzmann@siemens-healthineers.com (A.K.); michael.suehling@siemens-healthineers.com (M.S.); 6 Department of Radiology and Nuclear Medicine, University Medical Center Mannheim, 68167 Mannheim, Germany

**Keywords:** malignant lymphoma, splenic involvement, radiomics, machine learning, computer aided diagnosis, subtype classification, quantitative imaging biomarkers

## Abstract

**Simple Summary:**

In malignant lymphoma an early and accurate diagnosis is essential for therapy initiation and patient outcome. Within the diagnostic process, imaging plays a crucial role in disease staging. However, an invasive biopsy is required for subtype classification. Involvement of the spleen, a major lymphoid organ, is frequent in malignant lymphoma; this may be reactive or due to infiltration by malignant cells. Using radiomics features of the spleen in a machine learning approach, we investigated the possibility of distinguishing malignant lymphoma patients from other cancer patients and to classify lymphoma subtypes in the case of disease presence. Recent studies have proven the value of radiomics analysis in differentiating lymphoma from non-lymphoma groups on involved sites. Supported by machine learning, imaging could gain importance as a noninvasive diagnostic tool for future lymphoma classification, offering more precise radiological information for an interdisciplinary approach regarding treatment planning.

**Abstract:**

The spleen is often involved in malignant lymphoma, which manifests on CT as either splenomegaly or focal, hypodense lymphoma lesions. This study aimed to investigate the diagnostic value of radiomics features of the spleen in classifying malignant lymphoma against non-lymphoma as well as the determination of malignant lymphoma subtypes in the case of disease presence—in particular Hodgkin lymphoma (HL), diffuse large B-cell lymphoma (DLBCL), mantle-cell lymphoma (MCL), and follicular lymphoma (FL). Spleen segmentations of 326 patients (139 female, median age 54.1 +/− 18.7 years) were generated and 1317 radiomics features per patient were extracted. For subtype classification, we created four different binary differentiation tasks and addressed them with a Random Forest classifier using 10-fold cross-validation. To detect the most relevant features, permutation importance was analyzed. Classifier results using all features were: malignant lymphoma vs. non-lymphoma AUC = 0.86 (*p* < 0.01); HL vs. NHL AUC = 0.75 (*p* < 0.01); DLBCL vs. other NHL AUC = 0.65 (*p* < 0.01); MCL vs. FL AUC = 0.67 (*p* < 0.01). Classifying malignant lymphoma vs. non-lymphoma was also possible using only shape features AUC = 0.77 (*p* < 0.01), with the most important feature being sphericity. Based on only shape features, a significant AUC could be achieved for all tasks, however, best results were achieved combining shape and textural features. This study demonstrates the value of splenic imaging and radiomic analysis in the diagnostic process in malignant lymphoma detection and subtype classification.

## 1. Introduction

Malignant lymphomas represent a heterogenous group of neoplasms of lymphatic tissue and are divided into Hodgkin lymphoma (HL) and non-Hodgkin lymphoma (NHL). While many patients with malignant lymphoma present with rapid, indolent swelling of lymph nodes, the spleen as a major lymphoid organ is also often involved in the course of disease of these malignant processes. Splenic involvement is seen in approximately one third of all HL [1] at presentation and in varying proportions in other subtypes of NHL [2]. Splenomegaly alone has limited value as a sufficient biomarker for splenic involvement as 30% of normal-sized spleens can have focal tumor infiltration without splenomegaly [3,4]. Additionally, splenomegaly can occur without involvement in malignant lymphomas [4] and other causes of splenomegaly, such as infectious or autoimmune diseases are numerous.

Apart from histopathological classification according to the WHO classification of tumors of hematopoietic and lymphoid tissues, clinical imaging plays an important role in pretherapeutic staging and risk assessment of malignant lymphoma [5]. While PET-CT has some significant advantages to contrast-enhanced CT (ceCT) in detecting extranodal involvement, ceCT offers better accuracy in nodal size assessment [6,7]. Both HL and NHL are commonly staged using the Lugano classification of 2014 [7], which suggests a modified Ann Arbor staging system [8]. While splenic involvement is best determined by PET-CT, splenomegaly can be measured using a craniocaudal diameter (CCD) with a cut-off of more than 13 cm as proposed by the Lugano classification [7]. The CCD offers an easy-to-measure parameter to first assess potential splenic involvement in daily routine. We believe that the CCD and assessment of the entire spleen can be used for further analysis in lymphoma imaging. Quantitative imaging analysis techniques, including the field of radiomics, have shown in the past that a high-dimensional radiological feature set can correlate to pathophysiological aspects of cancer entities and can be linked to patient outcome [9,10,11,12,13,14].

This study evaluates whether quantitative imaging biomarkers (QIB) of the spleen can be used as a surrogate for predictions in the diagnostic process of malignant lymphoma. Therefore, we applied advanced machine learning techniques by using radiomics features of the spleen to explore the predictive value of the spleen for distinguishing malignant lymphoma patients from other cancer patients and to differentiate between malignant lymphoma subtypes, in particular, in HL and NHL subtypes: diffuse large B-cell lymphoma (DLBCL), follicular lymphoma (FL), and mantle-cell lymphoma (MCL). Recent studies on malignant lymphoma patients have shown the value of QIB analysis in distinguishing malignant lymphoma from other cancer entities [11,15,16,17,18]. Only a few studies further evaluated the potential benefit of radiomics analyses to distinguish between various malignant lymphoma subtypes [19,20].

While a differentiation of subtypes via noninvasive imaging will surely not replace histopathological analysis, it helps to better understand imaging characteristics of malignant lymphoma as complementary biomarkers and may offer more precise radiological information prior to, during, and after therapy.

The purpose of this study was to investigate the diagnostic value of radiomic features of the spleen in distinguishing malignant lymphoma from non-lymphoma as well as the determination of malignant lymphoma subtypes in the case of disease presence—in particular HL, DLBCL, MCL, and FL.

## 2. Materials and Methods

This study was approved by the Ethics Committee of the Ludwig Maximilians University of Munich and performed according to current guidelines for retrospective studies.

### 2.1. Patient Cohort and Non-Lymphoma Cohort

**Figure 1:**
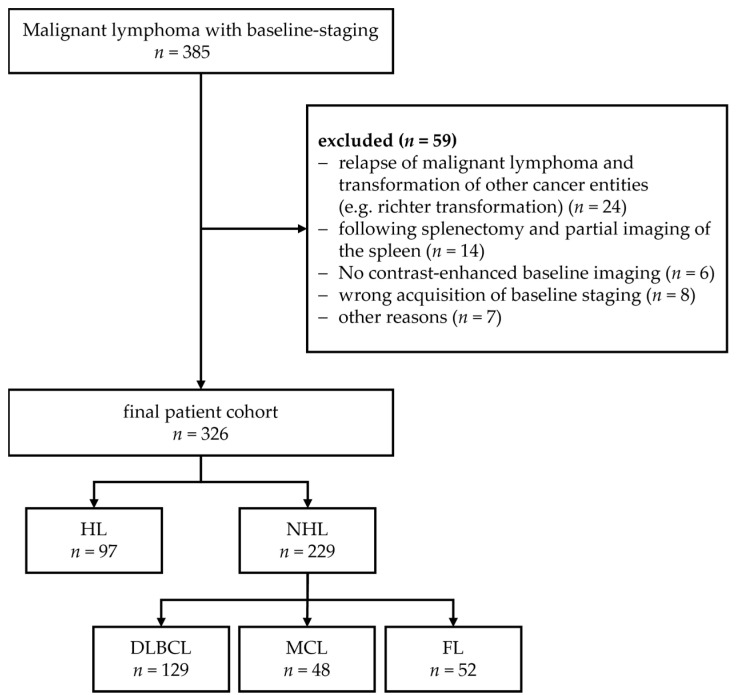
CONSORT diagram illustrating the final patient cohort of lymphoma patients and exclusion criteria.

We collected data from 385 patients with malignant lymphoma, who were treated in the University hospital Munich between March 2010 and March 2018. Data were retrieved as part of our inter-institutional study collaboration, which closed the study in March 2020. To be included in the study, patients had to be diagnosed with a histologically proven HL, DLBCL, FL, or MCL. Patients with a prior lymphoma or a primary CNS lymphoma were excluded. Furthermore, patients were excluded, if no ceCT staging was available, or no full image of the spleen was acquired. The final cohort consisted of 326 patients (Figure 1). For non-lymphoma patients, imaging data of patients with colorectal cancer were used. Spleen segmentations of a total of 56 patients were obtained for further analysis.

### 2.2. Imaging Data

The standard protocol for initial CT staging included a contrast-enhanced image series of the neck, chest, and abdomen. Images were acquired in the portal venous phase using 27 different multidetector-row CT scanners from 4 different vendors, of which the vast majority was acquired at the university hospital, nearly 40% of all scans were taken on a SOMATOM Definition Flash (Siemens Healthineers, Erlangen, Germany) in-house scanner. CeCT images were obtained by using a weight-adapted intravenous contrast agent. Chest images were obtained after 30 s; abdomen images were obtained after 70 s. For reconstruction, standard soft tissue kernels (I30f, B30f, Br36) were used. Slice thickness varied between 0.5 and 5 mm. To reflect the reality of lymphoma diagnosis in major lymphoma treatment centers, a heterogeneous image acquisition cohort was used to produce generalizable results and identify stable features. In daily clinical routine, patients either present themselves with preclinical imaging or receive a clinical and radiological workup during a hospital stay. All CT scans were reviewed by board-certified radiologists during clinical routine for stage of disease and splenic involvement. Radiological information about splenic involvement was retrieved from the radiology and nuclear medicine report whenever applicable, otherwise the images were reviewed again as part of this project to retrieve missing data. Screening of splenic involvement was performed by two board-certified radiologists in a blinded manner.

Images of the non-lymphoma cohort were acquired in portal venous phase using 17 different CT scanners from 3 different companies and included a ceCT of the abdomen in portal venous phase.

### 2.3. Image Segmentation

As a prerequisite for quantitative image analysis, the spleen was segmented on all CT scans. For 190 patients, this was done semi-automatically by board-certified radiologists in a custom software based on MeVisLab (MeVis Medical Solutions, Bremen, Germany; Fraunhofer MEVIS, Bremen, Germany) that offers automatic interpolation and refinement of contours to organ boundaries. A 3D neural network based on the U-Net [21] was then trained on these segmentations using deep learning. To ensure consistency of image segmentations, the U-Net was applied to both the remaining patients and the patients it was trained on. The results were carefully approved by board-certified radiologists and contours were corrected manually in rare cases if required.

### 2.4. Statistical Analysis

To summarize the data, descriptive statistics were used: dichotomous variables were stated in absolute frequency and percentage and were compared with the chi-squared test. Continuous data were tested for normal distribution with the Kolmogorov–Smirnov test and presented in median and lower and upper quartiles. To evaluate not normally distributed data the Mann–Whitney U-test was used.

### 2.5. Feature Extraction

To characterize the radiological appearance of the spleen, we computed all features available in the Python package PyRadiomics 3.0 [22], which provides a reference implementation of the features defined by the Image Biomarker Standardisation Initiative [23]. These first-order statistics, shape features, and different kinds of texture features were extracted on the original CT image, on eight Wavelet-filtered images, and on five LoG-filtered images (σ = 1, …, 5 mm). To the shape features, we added the CCD, defined as the extent of the spleen bounding box along the body axis, because it is the most common measure for spleen enlargement in the clinic [7]. Overall, we considered 1317 radiomics features.

To make the features from scans with different slice thicknesses comparable, all images were resampled to an isotropic voxel size of 1 mm prior to feature computation. To account for the heterogeneous CT scanners in our cohort, we investigated the effect of an additional feature harmonization using the ComBat method [24]. Batches were created by scanner vendor and slice thickness rounded to 1, …, 5 mm. Harmonization was applied to all features except shape features because the latter are directly computed from the segmentations and are therefore independent of the underlying image data.

To enable a better interpretability of the relevant features, we also investigated predictive models on subsets of these features: only 448 features on the original CT image, only 14 shape features (14), which are independent of variations in image acquisition, and, as baseline models reflecting clinical knowledge, CCD only and spleen volume only. Shape features including CCD and spleen volume are computed directly on the segmentations and not on the underlying image data. Therefore, these features are particularly suitable for a real-world data set like ours that is heterogeneous in terms of scanners and imaging settings.

### 2.6. Model Building and Analysis

For investigating associations between radiomics features of the spleen and the presence of lymphoma or the distinction of different lymphoma subtypes, we trained Random Forest classifiers in a 10-times repeated stratified 10-fold cross-validation. We used the Python package scikit-learn 0.24 [25] with default settings. We did not perform any feature scaling or selection because this is not required for the Random Forest classifier. Model quality was assessed using the mean area under the curve (AUC) over the folds and repetitions. Confidence intervals (CI) at 95% were computed by 100-times bootstrapping of the pooled out-of-sample predictions on patient level [26]. Models were considered significantly better than guessing when the 95% CIs of the AUC were completely above 0.5. Finally, to understand which features were most relevant for the classification, we trained a model on 80% of the data, this time with mRMR selection of 10 features to remove correlations and analyzed the permutation importance of the features with 100 permutations on the remaining 20%.

**Figure 2:**
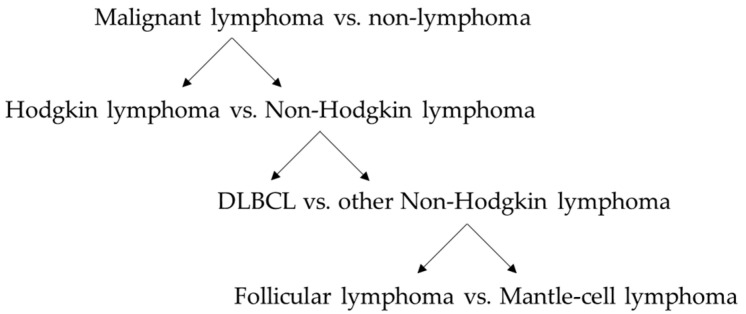
Cascade of binary classification tasks to distinguish malignant lymphoma and different lymphoma subtypes.

We applied this methodology to four different classification tasks, creating a cascade of binary classifiers to distinguish the five classes: (i) non-lymphoma, (ii) Hodgkin lymphoma, (iii) DLBCL, (iv) follicular lymphoma, and (v) mantle-cell lymphoma (Figure 2). To account for a potential bias by splenic involvement we analyzed the classification tasks on two cohorts: all patients and patients without diagnosed splenic involvement.

## 3. Results

### 3.1. Demographic Data

Baseline characteristics of the included patients are shown in Table 1. Overall, we retrieved imaging data from 326 patients, 125 (38.3%) ceCT images were available in a thin reconstruction (0.5 or 0.75 mm). The non-lymphoma cohort consisted of 56 patients with a median age of 61.5 years (SD 11.49 years), of which 35.7% were male. The patient cohort without splenic involvement consisted of 285 patients.

### 3.2. Segmentations of the Spleen

**Figure 3:**
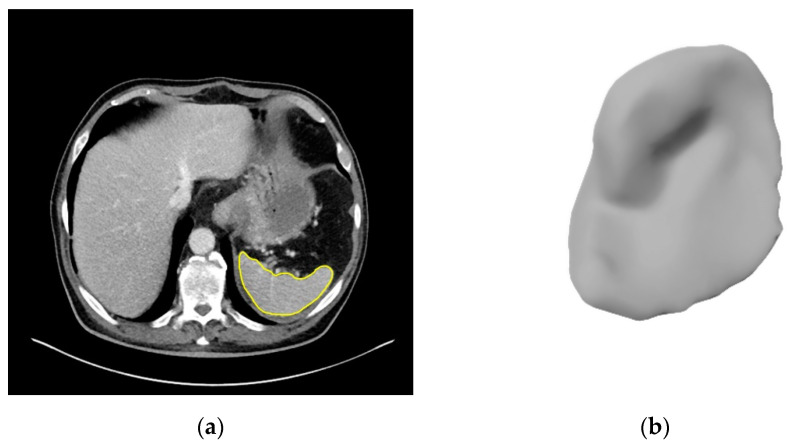

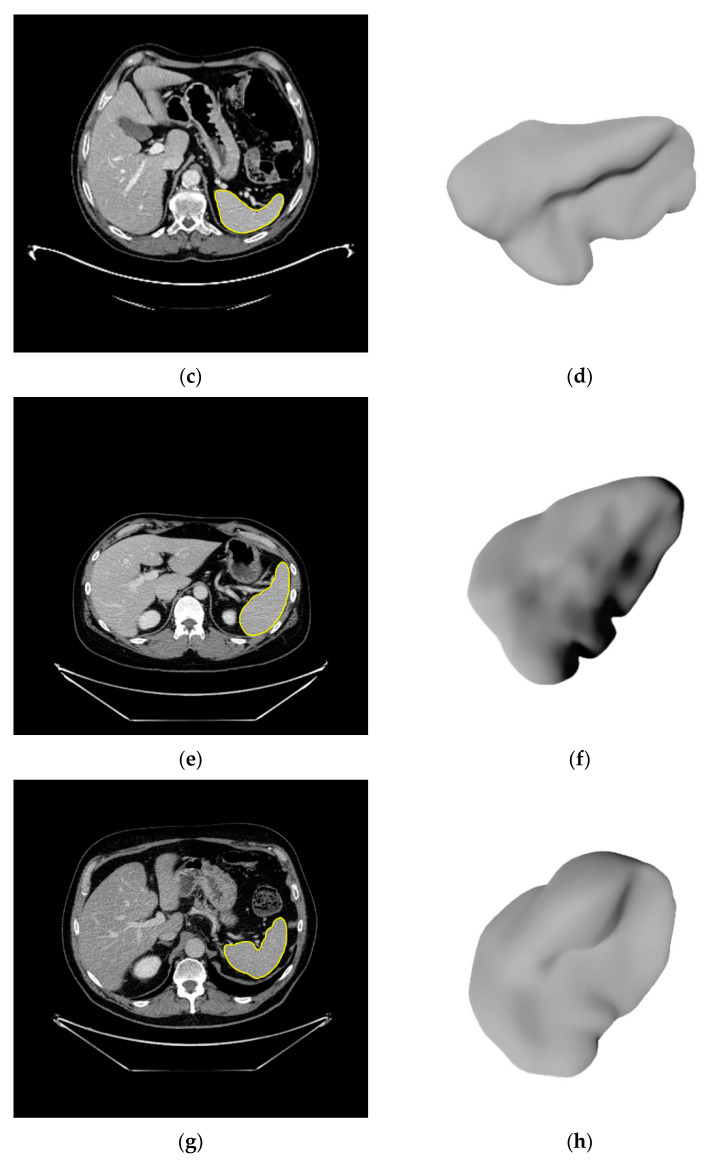

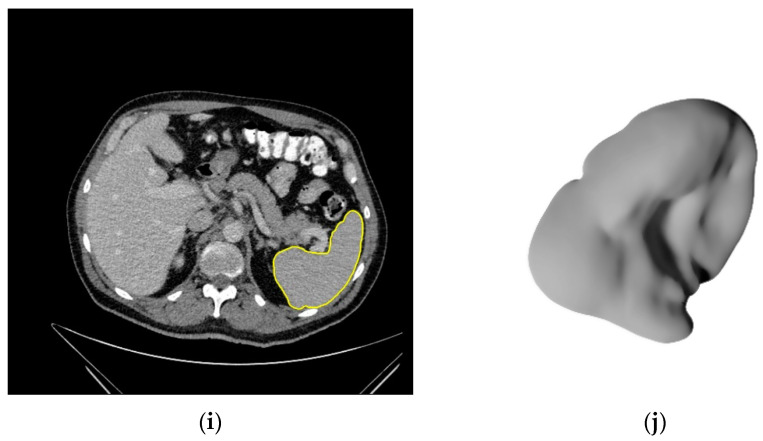
Example CT slices with splenic segmentation and 3D renderings of typical spleens: (**a**,**b**) non-lymphoma cohort, (**c**,**d**) Hodgkin lymphoma, (**e**,**f**) DLBCL, (**g**,**h**) follicular lymphoma, (**i**,**j**) mantle-cell lymphoma. For each type, we selected the spleen whose radiomics features were closest to the median of all spleens of that type.

In Figure 3, five representative patients are shown, visualizing a spleen whose radiomics features are closest to the median of the control cohort and each malignant lymphoma subtype, respectively.

### 3.3. Classifying Malignant Lymphoma vs. Non-Lymphoma

A Random Forest classifier for distinguishing all malignant lymphoma patients from controls based on features of the spleen achieved an AUC of 0.86 (CI: [0.80, 0.90]). The most important feature according to permutation importance is gray level non-uniformity on an image filtered with a LoG of σ = 3 mm, with an AUC decrease of 0.10. The AUC is almost the same when only using original features, but although sphericity is the most important feature, restricting the model to shape features leads to a drop of the AUC to 0.77 (CI: [0.70, 0.83]). Using CCD of the spleen as the only feature, malignant lymphoma patients are distinguished from controls with an AUC of 0.68 (CI: [0.61, 0.75]). For splenic volume in all patients, the AUC is 0.67 (CI: [0.60, 0.76]). After feature harmonization with ComBat, similar AUCs are achieved. The AUC when using original features is 0.81 vs. 0.85 but the CIs overlap and therefore the difference is not significant. The Random Forest classifiers were also applied to the subgroup of malignant lymphoma patients without splenic involvement, which produced similar AUCs as the classifiers on all patients. All results are summarized in Table 2.

### 3.4. Subtype Prediction in Malignant Lymphoma

The results for the cascade of classifiers predicting subtypes of malignant lymphoma are shown in Table 3, Table 4 and Table 5. For HL vs. NHL, the best AUC of all patients is 0.75 (CI [0.69, 0.81]) using all features, dropping to 0.65 (CI: [0.58, 0.71]) when using only original features (Table 3). For distinguishing DLBCL from other NHL in all patients, an AUC of 0.65 (CI: [0.56, 0.71]) is achieved with all features, but shape features are sufficient to reach an AUC of 0.62 (CI: [0.55, 0.68]) (Table 4). Finally, shape features alone yield the highest AUC of 0.71 (CI: [0.60, 0.80]) for FL vs. MCL in all patients, compared to 0.64 (CI: [0.54, 0.76]) with original features (Table 5). For none of the three tasks completed, classifiers using only volume or only CCD achieved an AUC above 0.6; significance over random was shown in only two of six cases in all patients. Again, the analysis was repeated with ComBat feature harmonization and the subgroup of patients without splenic involvement were analyzed separately. In both cases, similar results were achieved in nearly all classification tasks.

When using all features, the most important feature according to permutation importance was always a feature on a LoG-filtered image. In two cases, as well as in the lymphoma vs. non-lymphoma case, it is a texture feature. When using only features from the original image, the most important feature is always a shape feature, again consistent with lymphoma vs. non-lymphoma. Furthermore, shape features always perform comparably to original features.

## 4. Discussion

The goal of this study was to explore whether potentially robust predictors of the spleen for lymphoma detection and subtype classification could be identified based on quantitative imaging, which may serve as an imaging biomarker for noninvasive, early diagnostic directions in lymphoma patients. The results of this study suggest that (i) shape features of the spleen have more predictive value than the craniocaudal diameter of the spleen and spleen volume in detecting malignant lymphoma, (ii) radiomics features and subsets, like shape features, allow the differentiation of different malignant lymphoma subtypes, and (iii) the sphericity of the spleen is a key characteristic that has not gained a lot of scientific attention in malignant lymphoma classification until now. While there are several studies that used QIB for diagnostic classifications and predictions of patient outcome and prognosis in different cancer entities [9,13,28,29], only few have focused on malignant lymphoma [11,15,16,17]. Various studies focused on building a machine learning classification model significantly differentiating primary central nervous system lymphoma from glioblastoma [28,29]. Reinert et al. used a radiomics-based model to differentiate DLBCL Richter Transformation patients from chronic lymphocytic leukemia patients on ceCT images reaching an AUC of 0.85 [17]. In malignant lymphoma, a fast and precise diagnostic process is essential. Decisions regarding the therapy regimen and start of therapy are dependent on the subtype classification by the WHO [30] and on clinical imaging primarily for disease staging. All current methods of subtype classification are relying on invasive biopsies. The diagnostic standard is the extirpation of a whole involved lymph node, typically the easiest to biopsy, for pathological viewing and immunohistochemical staining. Histopathologic tissue analysis will doubtlessly remain the best diagnostic method in identifying malignant lymphoma subtypes. However, imaging methods can contribute complementary information throughout the patient journey and help to better understand malignant lymphoma dispersion in patients before, during, and after therapy.

Only few studies have focused on differentiating between lymphoma subgroups with imaging techniques, mostly focusing on involved lymph node sites. A recent study showed promising results in categorizing DLBCL and FL patients on MRI using statistical analysis correlating texture features and subtype [20]. Featuring the same subgroups as this study, Lippi et al. exploited a machine learning setting on texture analysis of involved lymph node sites in PET/CT images to further classify lymphoma [19]. One major time-consuming task in studies exploiting involved lymph node sites is the segmentation process, often requiring experts to manually segment volumes of interest. As intratumoral heterogeneity between involved sites has been demonstrated [31,32], imaging features can vary between involved sites [33], which is why choosing what sites to further exploit is a crucial step in those studies. In this study we focused on the spleen for two major reasons: firstly, malignant lymphoma is a systemic disease developing from lymphocytes. Therefore, the spleen as a major lymphoid organ is often involved in the process. Involvement of the spleen can be seen as primary or secondary involvement or as a reaction to a systemic lymphoid disease, without direct lymphoma manifestation in the spleen. We hypothesized that these reactions vary between lymphoma subgroups and can be represented using QIBs. Secondly, the spleen is a parenchymatous organ surrounded by peri-splenic fat and therefore easy to automatically segment. Humpire-Mamani et al. have shown that a neural network can be trained to segment the spleen in CT scans with an accuracy that is comparable to an experienced radiologist [34,35]. Such a performance would not be expected for involved lymph node sites, which can occur on any site within the scan volume. A recent study found significantly different CT-textural features in splenomegaly, differentiating splenic infiltration of lymphoma versus splenomegaly in liver cirrhosis and further exploiting their role in longitudinal lymphoma monitoring [36].

To evaluate the diagnostic value of the spleen in lymphoma patients for differentiating malignant lymphoma from non-lymphoma patients and classifying subtypes, segmentations of the spleen on baseline ceCT imaging were used to derive different QIBs. Extracted imaging features of the spleen included those from which Aerts et al. derived their established radiomics signature [9]. Meanwhile, this feature set has been extended and standardized [37] and the PyRadiomics package provides a reference implementation [22] that was used in this study. While these features include well-known parameters such as the mean density or the volume of a segmented structure, in particular, the texture features can be less intuitive. As an example, the gray level size zone matrix (GLSZM) non-uniformity, that is used by the most predictive feature for distinguishing lymphoma from non-lymphoma, measures whether some image values typically appear in larger connected areas than others, which would show as a particular pattern in the image. Such patterns can be unnoticeable to the human eye but still reflect properties of the visualized tissue [9,38,39].

In this study, we followed a classification tree (Figure 2) to assess binary classification tasks: to start with, we compared spleens of malignant lymphoma patients to spleens of non-lymphoma patients, as ceCT imaging is mostly the first imaging modality to be used to further explore an incidental finding or when symptoms occur. In the following step the differentiation of HL and NHL was addressed as they are historically divided into these subtypes by pathological appearance. For further classification of NHL subtypes, the aggressive subtype, DLBCL, was compared against other NHL. DLBCL is the most common subtype of NHL [40], matching our data set. Nearly equal sample sizes could be achieved by differentiating DLBCL against MCL and FL. The final step included classifying two indolent malignant lymphoma subtypes, FL to MCL. For classification, we applied machine learning to different feature sets, such as CCD and spleen volume and different types of radiomic features, to address each hypothesis.

The CCD of the spleen was used as a baseline to evaluate the binary classification tasks, as it represents a daily used splenic parameter and is easy to retrieve during clinical routine. Spleen volume on the other hand is unlikely to be retrieved in clinical routine but corresponds well with the CCD of the spleen for assessing splenomegaly [6,38]. By using an automated segmentation of the spleen, the exact volume could easily be extracted from images. While both CCD and volume could be used to significantly differentiate malignant lymphoma from the non-lymphoma cohort, the predictive value for further subtype differentiation was limited in following evaluations. That indicates an overall growth of spleens in lymphoma patients, but not necessarily above the cutoff for splenomegaly, as only 16.9% of included malignant lymphoma patients had a splenomegaly with a CCD > 13 cm. A classifier using shape features of the spleen produced a higher AUC = 0.77 (CI: [0.70, 0.83]) in differentiating malignant lymphoma patients and non-lymphoma patients, indicating that there is a benefit from other shape features as the spleen is not only increasing in size in malignant lymphoma patients but also changing its shape due to malignant lymphoma. Accordingly, the most important feature, when using only features from the original image for classifying malignant lymphoma against non-lymphoma, was a shape feature, the sphericity of the spleen. The sphericity describes the roundness of the shape of the spleen in relation to a sphere. As stated in the study by Reinert et al., it was possible to significantly differentiate splenomegaly in malignant lymphoma from splenomegaly in liver cirrhosis by only using textural features over time [36]. By combining shape and texture features we built a classification model with an AUC = 0.86 (CI: [0.80, 0.90]) for the first task.

Classifiers based on shape features could also be used to significantly differentiate between the varying subgroups of lymphoma, indicating that shape is not only different in malignant lymphoma vs. non-lymphoma patients but also varying between malignant lymphoma subtypes. Accordingly, the most important features, when using only features from the original image, were again shape features in all classification tasks. The best outcome for differentiating malignant lymphoma subtypes could be achieved in most of the binary classifiers using all available radiomic features, combining shape and texture features. It must be noted that numerically superior classifying results in differentiating the indolent subtypes MCL and FL were on the other hand achieved using only shape features. While the AUC is numerically higher than other classifiers, the CIs overlap and therefore the difference is not significant.

Direct splenic involvement of malignant lymphoma was observed in 12.6% of all patients in our cohort. In order to account for a potential bias in our analysis due to splenic involvement, we additionally analyzed a subgroup where we excluded patients with splenic involvement. In this subgroup, similar results as in the complete cohort were observed. These results suggest that other anatomical characteristics of the spleen are more relevant for the classification task. Overall, our results indicate that radiomic features of the spleen can serve as surrogates for CT-based lymphoma diagnosis and subtype differentiation. In order to extract this valuable information in CT scans, segmentations of the spleen are required. Currently, clinicians usually either do not have enough time or do not have the right tools to perform segmentations in their daily routine. In this study, a U-Net for automatic spleen segmentation was successfully trained with high accuracy. The U-Net is currently the state of the art for automatic image segmentation and was also used by Humpire-Mamani et al. for spleen segmentation [34]. When a U-Net is trained on scans from different scanners, with different slice thicknesses and so on, it learns a general concept of the appearance of a spleen that is independent of the actual imaging settings. Therefore, it performs robustly under a wide range of conditions. It is likely that new image viewing software will use these or similar technologies in the near future, leading to automated organ segmentations and therefore enabling radiologists to use inherent image information such as radiomics features in their clinical routine. The results of this study suggest that this could lead to an improved diagnostic assessment.

Our study has some potential limitations. As a retrospective, single-center study, we were able to retrieve a large sample size, however, when dividing our cohort into subgroups sample sizes varied between entities with DLBCL being the largest one and MCL patients being the smallest one with 48 patients. While there are many different subtypes of lymphoma, we only concentrated on four of the largest malignant lymphoma entities. As the non-lymphoma group, we used imaging data of patients with colorectal cancer, which of course does not necessarily indicate a healthy spleen, although splenic metastases in colorectal cancer are uncommon [39]. However, more subtle alterations of splenic size and shape cannot be definitely ruled out as cancer is a systemic disease with widespread effects on the whole body. The reason for choosing the colorectal cancer control group was mainly the similar scanning protocol and comparable scanning conditions at our center.

With 326 patients and images retrieved from 27 different CT scanners, images were acquired with a high variability in image acquisition. Especially textural features can be sensitive to image acquisition and reconstruction settings [41]. As the protocol suggests, ceCT images in portal venous phase, contrast enhancement can vary between patients due to contrast medium administration [42]. To ensure the validity of our results in this heterogeneous data set reflecting clinical reality, we additionally performed an analysis where the ComBat feature harmonization algorithm is applied to the extracted first-order and texture features. With this approach, we reduced potential effects of different scanner types and slice thicknesses on these features. However, we found that the AUCs remained very similar, indicating that the high degree of standardization in CT imaging is sufficient to make features comparable in a heterogeneous cohort for the given task.

As this study focuses solely on the spleen in differentiating lymphoma subtypes, additional information could be gained by analyzing other sites of disease such as enlarged lymph nodes. Further prospective studies are needed that combine the prognostic value of the spleen and other involved sites in malignant lymphoma patients.

Being able to differentiate malignant lymphoma subtypes from imaging data using radiomics analysis may be useful in the future for creating diagnostic support systems, benefiting the patient with a fast and interdisciplinary approach to a final diagnosis. While it will not replace the histopathological analysis of lymphoma, the diagnostic value of the spleen and other involved sites could become more important in noninvasive examination of lymphoma, offering more precise imaging information, and therefore assisting the pathologist in immunohistochemical decisions. In addition, it could also offer early valuable information on lymphoma progress and intratumoral mutations of genetic markers in lymphoma, which would have to be evaluated in further studies.

## 5. Conclusions

Imaging plays an important role in the diagnosis of lymphoma for defining the stage of disease, while the immunohistopathological workup of extracted lymph nodes defines the subtype of malignant lymphoma. The results of this study suggest that (i) shape features of the spleen have more predictive value than CCD and spleen volume in detecting malignant lymphoma, (ii) radiomics features allow the differentiation of different malignant lymphoma subtypes (HL, DLBCL, MCL, FL), and (iii) the sphericity of the spleen is a key characteristic that has not gained a lot of scientific attention in malignant lymphoma until now. The best results were achieved using all features to build classifiers. Classifiers using only shape features performed significantly as well, indicating that the shape of the spleen changes depending on lymphoma subtypes. The results of this study conclusively demonstrate that the spleen offers potential diagnostic value in lymphoma patients and should be evaluated in further studies.

## Figures and Tables

**Table 1:** Basic demographic data at initial diagnosis of the malignant lymphoma cohort and non-lymphoma cohort. Stages are defined following the Cotswold modification of the Ann Arbor staging system [27], the category “advanced disease” for initial staging of disease is defined by the Lugano classification system of 2014 [7].

Characteristics	HL	DLBCL	MCL	FL	Non-Lymphoma
Cases (percentage of cohort)	97 (29.8%)	129 (39.6%)	48 (14.7%)	52 (16%)	56
Age					
Median (years)	34.00	67.0	63.5	65.0	61.5
Lower/upper quartile (years)	25.0/45.0	50.0/74.5	51.0/68.8	53.3/71.0	51.3/67.0
Gender					
Male	49 (50.5%)	70 (54.3%)	34 (70.8%)	34 (65.4%)	20 (35.7%)
Female	48 (49.5%)	59 (45.7%)	14 (29.2%)	18 (34.6%)	36 (64.3%)
Stage (Ann Arbor)					
III/IV—“advanced disease”	38 (39.2%)	49 (38.0%)	40 (83.3%)	39 (75%)	-
IV	24 (24.7%)	31 (24.0%)	32 (66.7%)	21 (40.4%)	-
Craniocaudal diameter					
Median (mm)	101.60	93.00	131.50	101.20	89.50
Lower/upper quartile (mm)	90.00/116.25	77.15/110.70	100.00/189.00	91.88/119.80	76.50/101.75
Minimum–maximum (mm)	60.00–163.20	55.00–235.00	49.60–310.00	60.00–248.00	47.00–127.00
Splenic involvement	12 (12.4%)	10 (7.8%)	15 (31.6%)	4 (7.7%)	-

**Table 2:** Results for lymphoma vs. non-lymphoma classification with different feature sets. AUCs are given with 95% CI and marked with an asterisk (*) if the CIs are completely above 0.5.

Features	AUC [CI] All Patients	AUC [CI] All Patients +ComBat	AUC [CI] Patients without Splenic Involvement	Most Important Feature
All	0.86 * [0.80, 0.90]	0.85 * [0.80, 0.90]	0.85 * [0.79, 0.89]	log-sigma-3-0-mm-3D_glszm_GrayLevelNonUniformity
Original	0.85 * [0.78, 0.90]	0.81 * [0.74, 0.86]	0.83 * [0.78, 0.88]	original_shape_Sphericity
Shape	0.77 * [0.70, 0.83]	0.77 * [0.70, 0.83]	0.75 * [0.69, 0.80]	original_shape_Sphericity
Volume	0.67 * [0.60, 0.76]	0.67 * [0.60, 0.76]	0.65 * [0.58, 0.72]	-
CCD	0.68 * [0.61, 0.75]	0.68 * [0.61, 0.75]	0.67 * [0.59, 0.76]	-

**Table 3:** Results for Hodgkin vs. non-Hodgkin lymphoma classification with different feature sets. AUCs are given with 95% CI and marked with an asterisk (*) if the CIs are completely above 0.5.

Features	AUC [CI] All Patients	AUC [CI] All Patients +ComBat	AUC [CI] Patients without Splenic Involvement	Most Important Feature
All	0.75 * [0.69, 0.81]	0.75 * [0.69, 0.80]	0.73 * [0.65, 0.78]	log-sigma-5-0-mm-3D_firstorder_90Percentile
Original	0.65 * [0.58, 0.71]	0.65 * [0.58, 0.71]	0.63 * [0.57, 0.69]	original_shape_Maximum2DDiameterRow
Shape	0.61 * [0.54, 0.66]	0.61 * [0.54, 0.66]	0.63 * [0.56, 0.69]	original_shape_Sphericity
Volume	0.56 * [0.51, 0.61]	0.56 * [0.51, 0.61]	0.57 * [0.51, 0.63]	-
CCD	0.53 [0.46, 0.58]	0.53 [0.46, 0.58]	0.56 * [0.51, 0.62]	-

**Table 4:** Results for DLBCL vs. other non-Hodgkin lymphoma classification with different feature sets. AUCs are given with 95% CI and marked with an asterisk (*) if the CIs are completely above 0.5.

Features	AUC [CI] All Patients	AUC [CI] All Patients +ComBat	AUC [CI] Patients without Splenic Involvement	Most Important Feature
All	0.65 * [0.56, 0.71]	0.65 * [0.56, 0.71]	0.64 * [0.58, 0.70]	log-sigma-2-0-mm-3D_glrlm_RunEntropy
Original	0.63 * [0.55, 0.70]	0.63 * [0.55, 0.70]	0.66 * [0.60, 0.73]	original_shape_Maximum2DDiameterColumn
Shape	0.62 * [0.55, 0.68]	0.62 * [0.55, 0.68]	0.63 * [0.56, 0.69]	original_shape_Maximum2DDiameterColumn
Volume	0.52 [0.46, 0.59]	0.52 [0.46, 0.59]	0.53 [0.46, 0.61]	-
CCD	0.60 * [0.52, 0.66]	0.60 * [0.52, 0.66]	0.57 * [0.50, 0.63]	-

**Table 5:** Results for follicular vs. mantle-cell lymphoma classification with different feature sets. AUCs are given with 95% CI and marked with an asterisk (*) if the CIs are completely above 0.5.

Features	AUC [CI] All Patients	AUC [CI] All Patients +ComBat	AUC [CI] Patients without Splenic Involvement	Most Important Feature
All	0.67 * [0.55, 0.79]	0.67 * [0.55, 0.79]	0.65 * [0.53, 0.76]	log-sigma-5-0-mm-3D_glszm_SizeZoneNonUniformity
Original	0.64 * [0.54, 0.76]	0.65 * [0.54, 0.76]	0.64 * [0.52, 0.75]	original_shape_SurfaceVolumeRatio
Shape	0.71 * [0.60, 0.80]	0.71 * [0.60, 0.80]	0.69 * [0.60, 0.80]	original_shape_Flatness
Volume	0.59 [0.49, 0.70]	0.59 [0.49, 0.70]	0.58 [0.47, 0.70]	-
CCD	0.59 [0.46, 0.69]	0.59 [0.46, 0.69]	0.71 * [0.56, 0.83]	-

## Data Availability

Not applicable.
